# Added Prognostic Value of Hemorrhagic Transformation Quantification in Patients With Acute Ischemic Stroke

**DOI:** 10.3389/fneur.2020.582767

**Published:** 2020-11-10

**Authors:** Katinka R. van Kranendonk, Kilian M. Treurniet, Anna M. M. Boers, Olvert A. Berkhemer, Jonathan M. Coutinho, Hester F. Lingsma, Wim H. van Zwam, Aad van der Lugt, Robert J. van Oostenbrugge, Diederik W. J. Dippel, Yvo B. W. E. M. Roos, Henk A. Marquering, Charles B. L. M. Majoie, The MR CLEAN investigators

**Affiliations:** ^1^Department of Radiology and Nuclear Medicine, Amsterdam UMC, Location AMC, University of Amsterdam, Amsterdam, Netherlands; ^2^Department of Radiology, Haaglanden Medical Center (HMC), Den Haag, Netherlands; ^3^Nico.lab, Amsterdam, Netherlands; ^4^Department of Neurology, Erasmus MC-University Medical Center Rotterdam, Rotterdam, Netherlands; ^5^Department of Radiology & Nuclear Medicine, Erasmus MC-University Medical Center Rotterdam, Rotterdam, Netherlands; ^6^Department of Neurology, Amsterdam UMC, Location AMC, University of Amsterdam, Amsterdam, Netherlands; ^7^Department of Public Health, Center for Medical Decision Making, Erasmus MC-University Medical Center Rotterdam, Rotterdam, Netherlands; ^8^Department of Radiology and Nuclear Medicine, Cardiovascular Research Institute Maastricht (CARIM), University Medical Center, Maastricht, Netherlands; ^9^Department of Neurology, Cardiovascular Research Institute Maastricht (CARIM), Maastricht University Medical Center, Maastricht, Netherlands; ^10^Department of Biomedical Engineering and Physics, Amsterdam UMC, Location AMC, University of Amsterdam, Amsterdam, Netherlands

**Keywords:** ischemic stroke (IS), hemorrhagic transformation (HT), intracranial hemorrhage (ICH), endovascular therapy (EVT), hemorrhage volume, thrombolysis

## Abstract

**Introduction and Aim:** Hemorrhagic transformation (HT) frequently occurs after acute ischemic stroke and negatively influences the functional outcome. Usually, HT is classified by its radiological appearance. Discriminating between the subtypes can be complicated, and interobserver variation is considerable. Therefore, we aim to quantify rather than classify hemorrhage volumes and determine the association of hemorrhage volume with functional outcome in comparison with the European Cooperative Acute Stroke Study II classification.

**Patients and Methods:** We included patients from the MR CLEAN trial with follow-up imaging. Hemorrhage volume was estimated by manual delineation of the lesion, and HT was classified according to the European Cooperative Acute Stroke Study II classification [petechial hemorrhagic infarction types 1 (HI1) and 2 (HI2) and parenchymal hematoma types 1 (PH1) and 2 (PH2)] on follow-up CT 24 h to 2 weeks after treatment. We assessed functional outcome using the modified Rankin Scale 90 days after stroke onset. Ordinal logistic regression with and without adjustment for potential confounders was used to describe the association of hemorrhage volume with functional outcome. We created regression models including and excluding total lesion volume as a confounder.

**Results:** We included 478 patients. Of these patients, 222 had HT. Median hemorrhage volume was 3.37 ml (0.80–12.6) and per HT subgroup; HI1: 0.2 (0.0–1.7), HI2: 3.2 (1.7–6.1), PH1: 6.3 (4.2–13), and PH2: 47 (19–101). Hemorrhage volume was associated with functional outcome [adjusted common odds ratio (acOR): 0.83, 95% CI: 0.73–0.95] but not anymore after adjustment for total lesion volume (acOR: 0.99, 95% CI: 0.86–1.15, per 10 ml). Hemorrhage volume in patients with PH2 was significantly associated with functional outcome after adjusting total lesion volume (acOR: 0.70, 95% CI: 0.50–0.98).

**Conclusion:** HT volume is associated with functional outcomes in patients with acute ischemic stroke but not independent of total lesion volume. The extent of a PH2 was associated with outcome, suggesting that measuring hemorrhage volume only provides an additional benefit in the prediction of the outcome when a PH2 is present.

## Introduction

Hemorrhagic transformation (HT) commonly occurs as a natural progression or as a complication of reperfusion therapy for acute ischemic stroke (1, 2). Large, but also small HT subtypes were found to be associated with poor functional outcome (3). Incidence varies and differences in definition of HT between studies complicate comparisons between studies. Usually, HT is classified according to the European Cooperative Acute Stroke Study II (ECASS II) classification based on radiological appearance (4). This classification divides HT in four groups: hemorrhagic infarction type 1 (HI1), which is defined as small petechiae along the margins of the infarct; hemorrhagic infarction type 2 (HI2), defined as confluent petechiae within the infarcted area but no space-occupying effect; parenchymal hematoma type 1 (PH1) as blood clots in 30% or less of the infarcted area with some slight space-occupying effect; and parenchymal hematoma type 2 (PH2) as blood clots in more than 30% of the infarcted area with substantial space-occupying effect (4).

The ECASS classification only takes hemorrhage volume relative to the infarct volume into account when a PH is present, and therefore, small hemorrhages could be classified as PH2 when the infarct is small. The opposite is true when large hematomas develop within massive infarcts. These hematomas are not classified as PH2 when their relative size is <30% of the infarct while their objective size could be more than 40 ml. These hemorrhages might lead to symptomatic intracranial hemorrhage (sICH). However, according to the Heidelberg Bleeding classification, ICH other than PH2 might be symptomatic, but it is advised not to classify those hemorrhages as sICH (5).

Further, an agreement between observers for HT is only fair, as discriminating between HT subtypes can be challenging (6, 7). This limited agreement might contribute to a variation in the reported incidence of HT between studies.

As an alternative to the current rather crude classification of HT, we aim to quantify the hemorrhage volume of patients with HT and to assess its prognostic value by determining the association of hemorrhage volume with functional outcome in comparison with the ECASS II classification. Additionally, we determine whether hemorrhage volumes smaller than 30% of lesion volume might have been symptomatic.

## Methods

We included all patients with follow-up imaging from the MR CLEAN trial (8). The MR CLEAN trial was a multicenter randomized controlled trial that assessed the safety and efficacy of endovascular therapy compared with usual care after acute ischemic stroke due to large vessel occlusion. The MR CLEAN study protocol has been described previously (9).

We assessed potential HT on follow-up CT scans that were acquired ~5 days after inclusion. When these scans were not available, 24-h follow-up CT scans were examined. Hemorrhage volume was measured by a trained observer (KRK) by manually delineating the hemorrhages using ITK-SNAP (version 3.4.0). Hemorrhage volume consists of all hemorrhage present on the CT scan, including concomitant intraventricular hemorrhage and subarachnoid hemorrhage. HT was classified according to the ECASS II classification (4). In the MR CLEAN trial, sICH was classified as neurologic deterioration with an increase of more than four points on the National Institute of Health Stroke Scale and hemorrhage visible on imaging (8).

The functional outcome was assessed at ~90 days after stroke onset and attributed with a score according to the modified Rankin Scale (mRS). The mRS ranges from 0 to 6, where 0 indicates no symptoms and 6 indicates death.

### Statistical Analysis

Mean and SD are used to summarize normally distributed variables; for non-normal distributed variables, the median and interquartile range are used. We compared hemorrhage volumes between all HT subtypes using a Kruskal–Wallis test. The association of hemorrhage volume with functional outcome was assessed using ordinal logistic regression analysis using the full mRS scale as the outcome measure. The association of hemorrhage volume with functional outcome was estimated as a common odds ratio (cOR) per 10-ml increase, expressing the relative risk of a shift in the direction of good outcomes for every 10 ml of hemorrhage. A cOR < 1 indicates a shift toward worse outcomes on the mRS. Three models were made; in the first model, we assessed the association of hemorrhage volume with functional outcome. In the second model, we assessed the association of hemorrhage volume and all HT subgroups with functional outcome, and the third model described the association of hemorrhage volume and sICH with functional outcome. We adjusted every model for potential confounders: diabetes mellitus, systolic blood pressure (measured on admission), intravenous thrombolysis, endovascular therapy, time from onset to randomization, history of ischemic stroke, age, atrial fibrillation, and baseline National Institutes of Health Stroke Scale. We conducted an additional subgroup analysis to assess the association of hemorrhage volume with functional outcome per HT subgroup.

Follow-up lesion volume included both infarct and hemorrhage volume and was estimated using a validated automated measurement (10). In some patients with a large PH, the lesion volume is equal to the hemorrhage volume, and the actual infarct is masked by hemorrhage. Adjusting for follow-up lesion volume might result in an underestimation of the impact of hemorrhage volume. However, HT is more likely to occur within large infarcts, and not adjusting for lesion volume could overestimate the impact of HT. Therefore, we conducted analyses with additional adjustment for follow-up lesion volume. We conducted the statistical analysis using R {R Core Team [V.4.0.0 (2020)]; R: A language and environment for statistical computing, R Foundation for Statistical Computing, Vienna, Austria; used packages rms (11), ggplot2 (12), and tableone (13)}.

## Results

**Figure 1:**
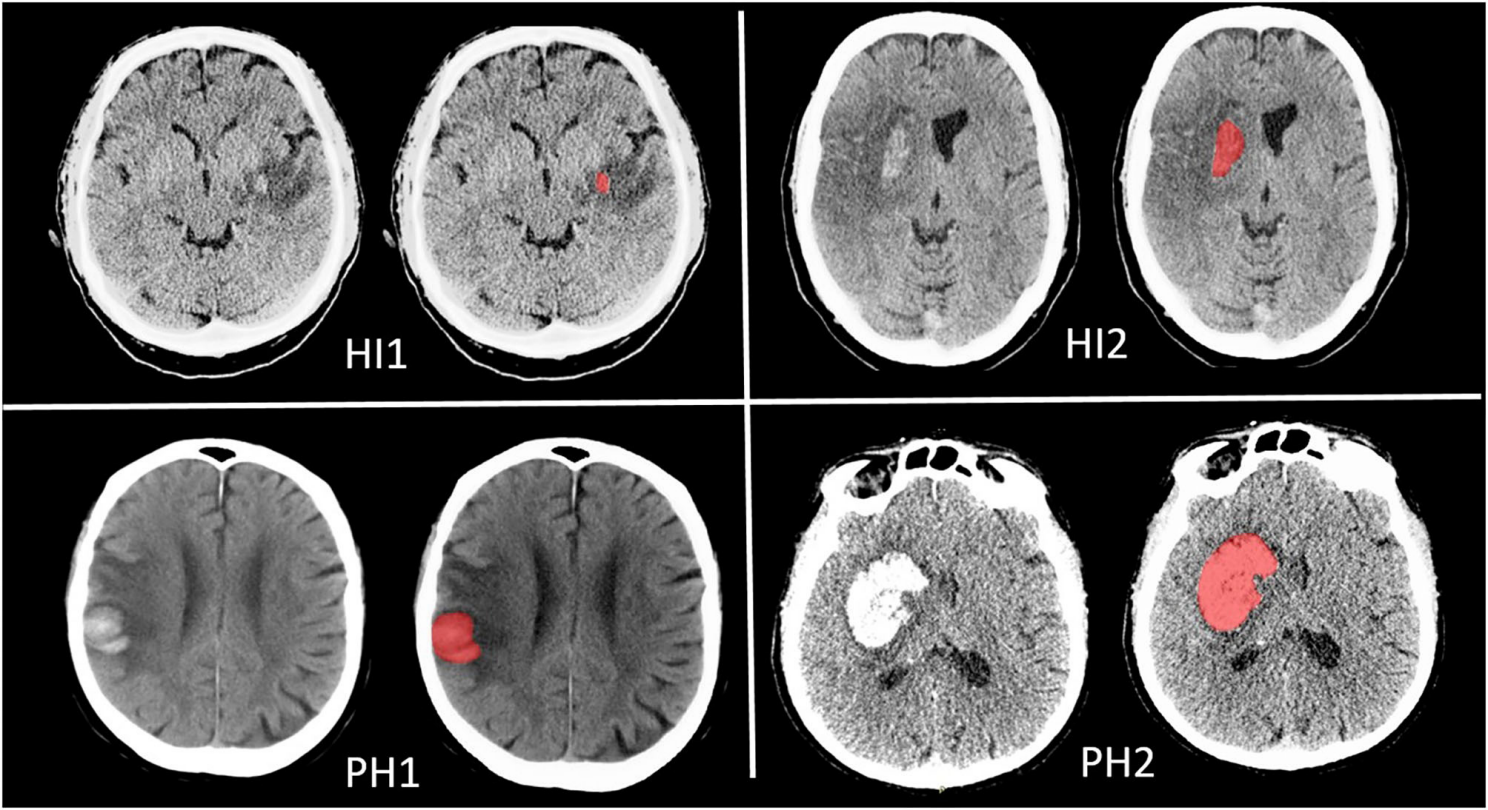
Quantification of Hemorrhagic Transformation per subtype.

Of all the patients with follow-up imaging (*n* = 478), 222 had HT. Of these 222 patients with HT, we measured hemorrhage volumes of 219 patients (Figure 1). Hemorrhage volumes of three patients could not be measured due to insufficient image quality.

Hemorrhage volumes differed between HT subgroups (*p* < 0.001). Patients with PH2 had the largest hemorrhage volumes [46.8 (interquartile range: 19–101) ml] (Table 1).

**Table 1:** Patient characteristics.

	**No HT** **(*n* = 256)**	**HI1** **(*n* = 76)**	**HI2** **(*n* = 71)**	**PH1** **(*n* = 36)**	**PH2** **(*n* = 39)**
Hemorrhage volume, ml—median (IQR)	0 (0–0)	0.17 (0–1.7)	3.2 (1.7–6.1)	6.3 (4.2–12.9)	46.8 (18.7–100.7)
EVT—no. (%)	111 (43.4)	28 (36.8)	35 (49.3)	21 (58.3)	17 (43.6)
Treatment with IV alteplase—no. (%)	228 (89.1)	66 (86.8)	65 (91.5)	33 (91.7)	36 (92.3)
Age—mean (SD)	64 (14.3)	65 (12.6)	66 (12.9)	64 (14.8)	68 (14.1)
Baseline NIHSS—mean (SD)	17 (5.7)	19 (6.1)	18 (4.4)	18 (4.3)	19 (4.8)
History of ischemic stroke—no. (%)	26 (10.2)	7 (9.2)	8 (11.3)	1 (2.8)	9 (23.1)
Atrial fibrillation—no. (%)	58 (22.7)	17 (22.4)	25 (35.2)	15 (41.7)	14 (35.9)
Diabetes mellitus—no. (%)	27 (10.5)	13 (17.1)	9 (12.7)	5 (13.9)	7 (17.9)
Systolic blood pressure—mean (SD)	143 (22.4)	144 (26)	142 (27.5)	153 (23.6)	160 (31.4)
Time from stroke onset to randomization per minute—median (IQR)	193 (147–254)	217 (148–258)	207 (158–281)	213 (165–278)	223 (181–265)
Follow-up lesion volume—median (IQR)	47 (18–118)	132 (58–207)	120 (78–243)	172 (97–274)	165 (93–323)

*EVT, endovascular treatment; HT, hemorrhagic transformation; HI, hemorrhagic infarction; PH, parenchymal hematoma; NIHSS, National Institute Of Health Stroke Scale; IQR, interquartile range*.

**Figure 2:**
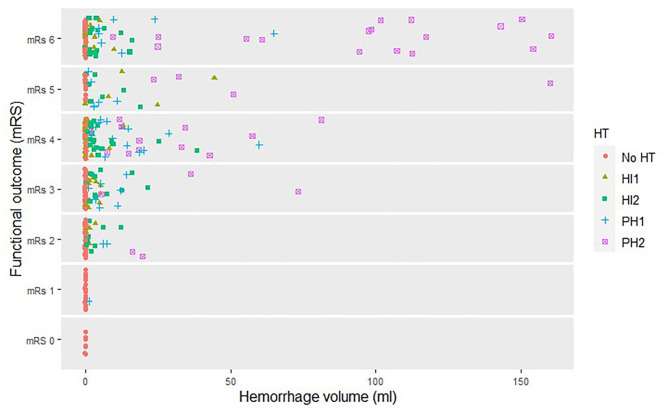
Hemorrhage volume and functional outcome per HT subgroup.

Hemorrhage volume was significantly associated with worse functional outcomes in the unadjusted and adjusted analyses [cOR 0.75, 95% confidence interval (CI) 0.67 to 0.83 and acOR 0.77, 95% CI: 0.69 to 0.87 per 10 ml] (Figure 2). After additional adjustment for follow-up lesion volume, the association was weaker (acOR 0.90, 95% CI 0.80 to 1.02) (Table 2, Model 1).

**Table 2:** Adjusted and unadjusted OR's of the association of hemorrhage volume in ml with functional outcome.

**Model**		**Unadjusted OR and 95% CI**	**Adjusted OR and 95% CI**	**Adjusted OR and 95% CI (with FLV)**
1	Hemorrhage volume, per 10 ml	0.75 (0.67 to 0.83)	0.77 (0.69 to 0.87)	0.90 (0.80 to 1.02)
2	Hemorrhage volume, per 10 ml	0.79 (0.69 to 0.90)	0.83 (0.73 to 0.95)	0.99 (0.86 to 1.15)
	HI1	0.42 (0.27 to 0.65)	0.56 (0.36 to 0.89)	0.68 (0.42 to 1.06)
	HI2	0.40 (0.24 to 0.64)	0.44 (0.27 to 0.71)	0.58 (0.34 to 0.96)
	PH1	0.46 (0.25 to 0.85)	0.41 (0.22 to 0.78)	0.72 (0.37 to 1.41)
	PH2	0.55 (0.23 to 1.31)	0.50 (0.20 to 1.24)	0.37 (0.14 to 0.98)
3	Hemorrhage volume, per 10 ml	0.83 (0.73 to 0.95)	0.83 (0.73 to 0.95)	0.94 (0.80 to 1.09)
	sICH	0.31 (0.12 to 0.78)	0.45 (0.17 to 1.17)	0.69 (0.23 to 2.07)

*The association of hemorrhage volume in milliliters with the full scale mRS score*.

*This table lists the association of hemorrhage volume with the full scale mRS score*.

*Adjusted for HT classification, atrial fibrillation, baseline NIHSS, intravenous thrombolysis, diabetes mellitus, time from stroke onset to randomization, age, endovascular therapy, previous stroke, systolic blood pressure (measured on admission). An additional analysis was conducted with follow-up lesion volume included in the adjusted analysis*.

*In models 2 and 3, no HT and no sICH were used as reference level when assessing the association of HT subgroups and sICH with functional outcome*.

*FLV, Follow-up lesion volume; HT, hemorrhagic transformation; HI, hemorrhagic infarction; PH, parenchymal hematoma; sICH, symptomatic intracranial hemorrhage; mRS, modified Rankin Scale*.

In model 2, the analysis that included hemorrhage volume and all HT subgroups, hemorrhage volume and all HT subgroups except PH2 with no HT as reference level were significantly and independently associated with functional outcome in the adjusted and unadjusted analyses. After additional adjustment for follow-up lesion volume, only HI2 and PH2 were associated with functional outcome (acOR 0.57, 95% CI 0.34 to 0.95 and acOR 0.36, 95% CI 0.14 to 0.97, respectively).

### Subgroup Analysis

**Figure 3:**
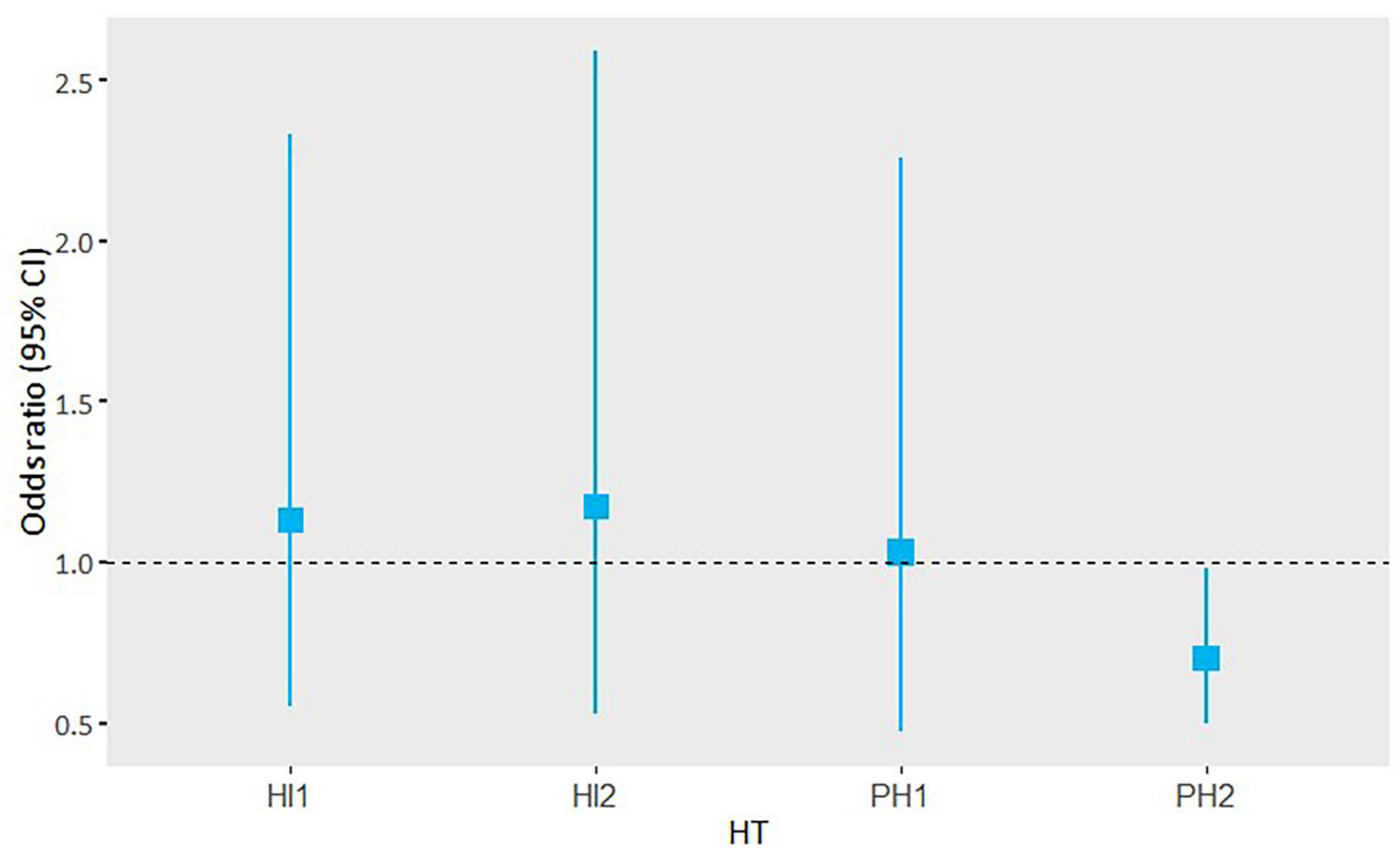
Adjusted OR and 95% CI of the subgroup analysis of hemorrhage volume and its association with functional outcome per HT subgroup.

Hemorrhage volume in patients with PH2 was significantly associated with functional outcome in the adjusted analysis, including follow-up lesion volume (acOR 0.70, 95% CI 0.50 to 0.98). This association was not observed in the other HT subtypes (Figure 3).

### Symptomatic Intracranial Hemorrhage

**Figure 4:**
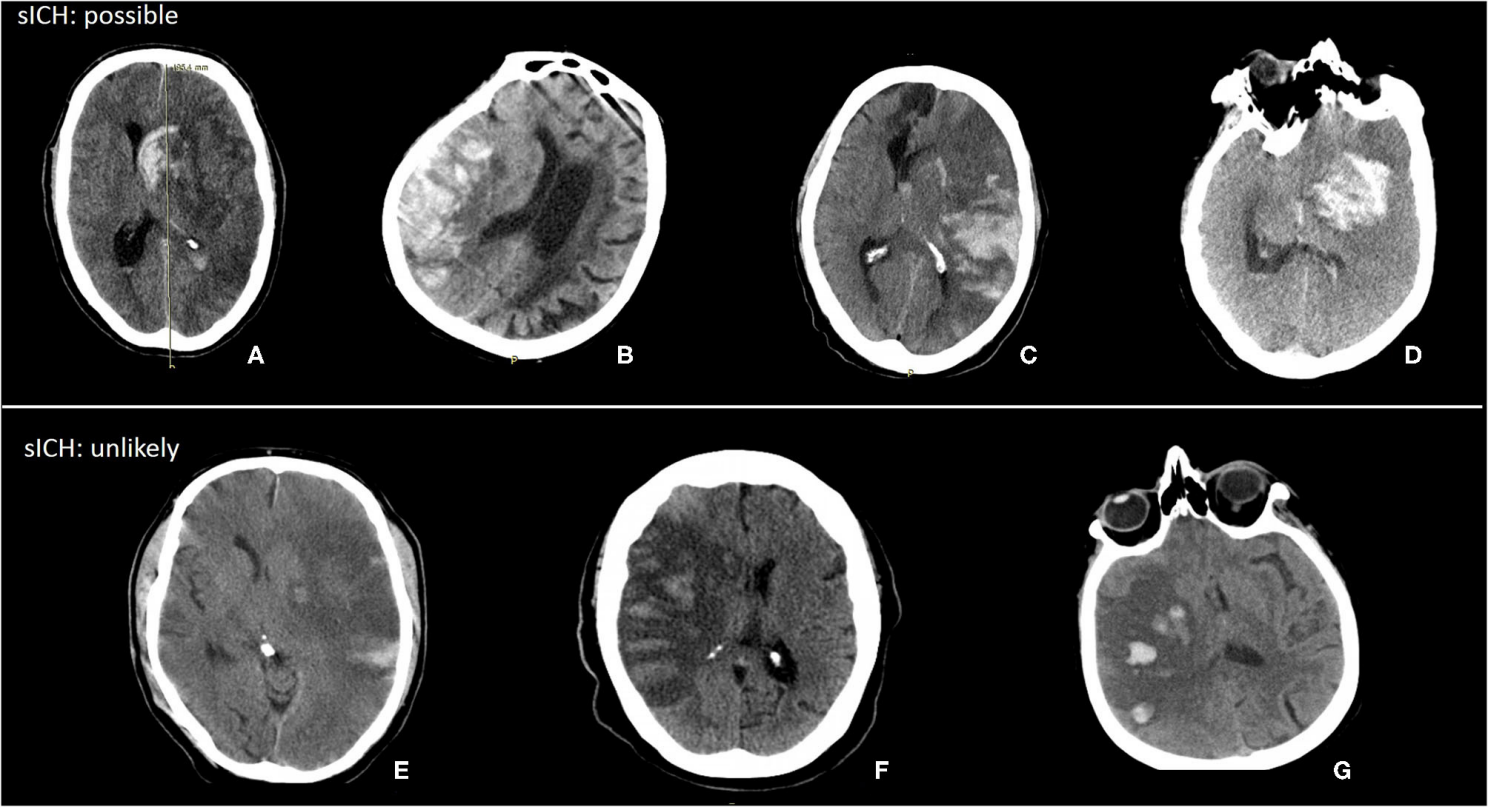
Examples of possible and unlikely sICH with hemorrhage volume < 30% of lesion volume. **(A)** Hemorrhage volume: 65 ml, lesion volume: 279 ml, hemorrhage (%): 24%. **(B)** Hemorrhage volume: 61 ml, lesion volume: 275 ml, hemorrhage (%): 22%. **(C)** Hemorrhage volume: 102 ml, lesion volume: 423 ml, hemorrhage (%): 24%. **(D)** Hemorrhage volume: 51 ml, lesion volume: 347 ml, hemorrhage (%): 15%. **(E)** Hemorrhage volume: 5 ml, lesion volume: 411 ml, hemorrhage (%): 1%. **(F)** Hemorrhage volume: 13 ml, lesion volume: 215 ml, hemorrhage (%): 6%. **(G)** Hemorrhage volume: 12 ml, lesion volume: 191 ml, hemorrhage (%): 6%.

Thirty-five patients with HT were classified as sICH (example in Figure 4). The median hemorrhage volume of patients with sICH was 53 (24–106) ml. Of those patients with sICH, 14 had hemorrhages <30% of lesion volume. Some of these hemorrhages likely caused symptoms, whereas in some sICH, not only hemorrhage would have caused symptoms but also infarct growth probably contributed to the neurological deterioration.

In model 3, hemorrhage volume and sICH were both significantly associated with functional outcome (cOR 0.83, 95% CI 0.73 to 0.95) and the unadjusted analysis (cOR 0.31, 95% CI 0.12 to 0.78). In the adjusted analyses, sICH was not significantly associated with functional outcome (acOR 0.45, 95% CI 0.17 to 1.17) (Table 2). After additional adjustment for follow-up lesion volume, the association of hemorrhagic volume with functional outcome was attenuated.

## Discussion

We have shown that hemorrhage volume and the ECASS classification are associated with functional outcomes independently of each other. In the adjusted analysis, hemorrhage volume was associated with functional outcome, and PH2 was not associated with functional outcome. This was also seen in the analysis with sICH. However, in the adjusted analysis that included follow-up lesion volume, hemorrhage volume was not associated with functional outcome. In the subgroup analysis, only hemorrhage volume of PH2 was associated with functional outcome.

Previous studies suggested that hemorrhage volume could be more appropriate than a radiological classification, as it gives a more objective description when assessing HT (14, 15). These studies had a relatively small sample size compared with the sample size of our study. Moreover, they only included patients with PH. In our study, we have shown that patients with large PH2 are more likely to have poor functional outcomes. This effect was not seen in the smaller HT subtypes (HI1, HI2, and PH1). This suggests that hemorrhage volume has only prognostic value in patients with a PH2.

Assessing the “true” effect of hemorrhage on outcome warrants adjustment for final infarct size due to their association. However, the definition of follow-up lesion volume as used in major studies (a combination of final infarct volume, swelling, edema, and hemorrhage) causes some difficulty (16). In patients with HI, adjustment for follow-up lesion volume will likely result in an accurate estimate of outcome. Quantifying these hemorrhages can be complicated, as the delineation of the hemorrhage is not clear. The brain tissue can be swollen and have petechial bleedings. Delineating the petechial bleedings results in small hemorrhage volumes, and the impact of the swollen brain tissue is not taken into account in this assessment. The lack of including a measure for swelling can result in a stronger observed association of the HI1, HI2, and PH1 classifications with the functional outcome than hemorrhage volume alone. However, large lesion volumes (incorporating both infarct and parenchymal swelling) are associated with HT and with a poor functional outcome, prompting us to include follow-up lesion volume in the analysis (17). As this measure includes hemorrhage, infarcted tissue, and edema while the proportion of hemorrhage is small, it will be correct to adjust for lesion volume. Conversely, for patients with a PH, hemorrhages can be large and tend to mask the infarct volume completely. In these cases, the value of the lesion volume is similar to the hemorrhage volume. Adding both values to the analysis will underestimate the association of large hemorrhage volumes with functional outcome.

Not all patients with sICH have a PH2 with a hematoma that consists of more than 30% of the infarct volume. When the infarct is very large, even a hemorrhage of 100 ml is <30% of the infarct volume, but it might cause symptoms and neurologic deterioration. However, some of the patients classified with sICH were unlikely to have symptoms due to hemorrhage. In almost all examples of sICH we showed, a midline shift was present. In four cases, the hemorrhage might have contributed to the midline shift leading to poor functional outcomes. In the other three cases, the midline shift was probably caused by infarct growth and not due to hemorrhage. For the classification of sICH, it is important to determine if it is likely that the hemorrhage is causing the symptoms as has been proposed in the Heidelberg Bleeding Classification (5).

An advantage of quantifying hemorrhage volume is that it might be less sensitive to interobserver variability than classifying HT. Quantifying hemorrhage volume can be time-consuming. However, it may be possible to automatically quantify hemorrhage volume, as this is accomplished with subarachnoid hemorrhage and hemorrhagic stroke (18, 19). In some HT cases, it is difficult to distinguish petechial hemorrhage from remaining intact cortex throughout the infarct, also introducing a subjective element when performing a manual assessment. Making it more accessible and less sensitive to interobserver variability when classifying HT, it could be classified as HT or no HT and measure hemorrhage volume only when a PH is present.

This study had several limitations; some patients had diffuse brain swelling with hemorrhage, which is complicated to delineate and could have resulted in smaller hemorrhage volumes for those patients. Hemorrhage volumes were quantified by one observer, and therefore, the interobserver variability could not be assessed. However, measuring hemorrhage volume by one observer leads to less variation to assess its association with functional outcome, and eventually, hemorrhage volume might be assessed by an automated measurement.

In conclusion, hemorrhage volume is associated with functional outcomes but not independent of total lesion volume. However, hemorrhage volume could be useful for classifying HT particularly to measure the extent of a PH.

## Data Availability Statement

The datasets presented in this article are not readily available because of their sensitive nature. Requests to access the datasets should be directed to the MR CLEAN executive committee (mrclean-trial.org).

## Ethics Statement

The studies involving human participants were reviewed and approved by a central medical ethics committee and the research boards of all participating centers accepted the MR CLEAN trial. The patients/participants provided their written informed consent to participate in this study.

## Author Contributions

WZ, AL, RO, DD, YR, and CM designed the MR CLEAN trial. OB collected and prepared the data for the trial. KK, OB, AB, and KT prepared data for this study. KK performed the statistical analysis, interpreted the results, and drafted the paper. HM, KT, and CM assisted with the statistical analysis, interpretation of the results, and drafting the paper. OB, HL, WZ, AL, RO, DD, YR, and CM critically revised the paper. All authors contributed to the article and approved the submitted version.

## Conflict of Interest

Academic medical center Amsterdam received funds from Stryker for consultations by CM, YR, and OB. CM received research grants from Cardiovasculair Onderzoek Nederland (CVON)/Dutch Heart Foundation, European Commission, Twin Foundation and Stryker (all paid to institution). HM, AB, CM, and YR are shareholders of Nico.lab, a company that focuses on the use of artificial intelligence for medical image analysis. Erasmus University Medical Center received funds from Bracco Imaging for consultations by DD. Erasmus University Medical Center received funds from CVON/Dutch Heart Foundation, European Commission, Stryker, Penumbra, and Medtronic for the execution of stroke trials by DD and AL. Maastricht University Medical Center received funds from Stryker and Cerenovus for consultations by WZ. The remaining authors declare that the research was conducted in the absence of any commercial or financial relationships that could be construed as a potential conflict of interest.

## MR CLEAN investigators

Olvert A. Berkhemer, Amsterdam UMC, location AMC, the Netherlands and Erasmus MC-University Medical Center Rotterdam, the Netherlands. Puck S.S. Fransen, Erasmus MC-University Medical Center Rotterdam, the Netherlands. Debbie Beumer, Erasmus MC-University Medical Center Rotterdam, the Netherlands and Maastricht University Medical Center and Cardiovascular Research Institute Maastricht (CARIM), the Netherlands. Berkhemer, Fransen, and Beumer contributed equally. Lucie A. van den Berg, Amsterdam UMC, location AMC, the Netherlands. Hester F. Lingsma, Erasmus MC-University Medical Center Rotterdam, the Netherlands. Albert J. Yoo, Massachusetts General Hospital, Boston, United States of America. Wouter J. Schonewille, Saint Antonius Hospital, Nieuwegein, the Netherlands. Jan Albert Vos, MD, Sint Antonius Hospital, Nieuwegein, the Netherlands. Paul J. Nederkoorn, Amsterdam UMC, location AMC, the Netherlands. Marieke J.H. Wermer and Marianne A.A. van Walderveen, Leiden University Medical Center, the Netherlands. Julie Staals, Maastricht University Medical Center and Cardiovascular Research Institute Maastricht (CARIM), the Netherlands. Jeannette Hofmeijer and Jacques A. van Oostayen, Rijnstate Hospital, Arnhem, the Netherlands. Geert J. Lycklama à Nijeholt and Jelis Boiten, MC Haaglanden, the Hague, the Netherlands. Patrick A. Brouwer and Bart J. Emmer, Erasmus MC-University Medical Center Rotterdam, the Netherlands. Sebastiaan F. de Bruijn and Lukas C. van Dijk, HAGA Hospital, the Hague, the Netherlands. L. Jaap Kappelle, University Medical Center Utrecht, the Netherlands. Rob H. Lo, University Medical Center Utrecht, the Netherlands. Ewoud J. van Dijk and Joost de Vries, Radboud University Medical Center, Nijmegen, the Netherlands. Paul L.M. de Kort and Willem Jan J. van Rooij, Sint Elisabeth Hospital, Tilburg, the Netherlands. Jan S.P. van den Berg and Boudewijn A.A.M. van Hasselt, Isala Klinieken, Zwolle, the Netherlands. Leo A.M. Aerden and René J. Dallinga, Reinier de Graaf Gasthuis, Delft, the Netherlands. Marieke C. Visser and Joseph C.J. Bot, Amsterdam UMC, location VU, Amsterdam, the Netherlands. Patrick C. Vroomen and Omid Eshghi, University Medical Center Groningen, the Netherlands. Tobien H.C.M.L. Schreuder and Roel J.J. Heijboer, Atrium Medical Center, Heerlen, the Netherlands. Koos Keizer and Alexander V. Tielbeek, Catharina Hospital, Eindhoven, the Netherlands. Heleen M. den Hertog and Dick G. Gerrits, Medical Spectrum Twente, Enschede, the Netherlands. Renske M. van den Berg-Vos and Giorgos B. Karas, Sint Lucas Andreas Hospital, Amsterdam, the Netherlands. Ewout W. Steyerberg, Erasmus MC-University Medical Center Rotterdam, the Netherlands. H. Zwenneke Flach, Isala Klinieken, Zwolle, the Netherlands. Henk A. Marquering and Marieke E.S. Sprengers, Amsterdam UMC, location AMC, the Netherlands. Sjoerd F.M. Jenniskens, Radboud University Medical Center, Nijmegen, the Netherlands. Ludo F.M. Beenen and René van den Berg, Amsterdam UMC, location AMC, the Netherlands. Peter J. Koudstaal and Wim H. van Zwam, Erasmus MC-University Medical Center Rotterdam, the Netherlands. Yvo B.W.E.M. Roos, Amsterdam UMC, location AMC, the Netherlands. Aad van der Lugt, Erasmus MC-University Medical Center Rotterdam, the Netherlands. Robert J. van Oostenbrugge, Maastricht University Medical Center and Cardiovascular Research Institute Maastricht (CARIM), the Netherlands. Charles B.L.M. Majoie, Amsterdam UMC, location AMC, the Netherlands. Diederik W.J. Dippel, Erasmus MC-University Medical Center Rotterdam, the Netherlands. van Zwam, Roos, van der Lugt, van Oostenbrugge, Majoie and Dippel contributed equally.
